# Characterization of seed nuclei in glucagon aggregation using light scattering methods and field-flow fractionation

**DOI:** 10.1186/1754-1611-2-10

**Published:** 2008-07-09

**Authors:** Cindy C Hoppe, Lida T Nguyen, Lee E Kirsch, John M Wiencek

**Affiliations:** 1 Department of Chemical & Biochemical Engineering, University of Iowa, Iowa City, IA, 52242, USA; 2 Division of Pharmaceutics, University of Iowa, Iowa City, IA, 52242, USA; 3 Department of Chemical Engineering, University of South Florida, Tampa, FL, 33520, USA

## Abstract

**Background:**

Glucagon is a peptide hormone with many uses as a therapeutic agent, including the emergency treatment of hypoglycemia. Physical instability of glucagon in solution leads to problems with the manufacture, formulation, and delivery of this pharmaceutical product. Glucagon has been shown to aggregate and form fibrils and gels *in vitro*. Small oligomeric precursors serve to initiate and nucleate the aggregation process. In this study, these initial aggregates, or seed nuclei, are characterized in bulk solution using light scattering methods and field-flow fractionation.

**Results:**

High molecular weight aggregates of glucagon were detected in otherwise monomeric solutions using light scattering techniques. These aggregates were detected upon initial mixing of glucagon powder in dilute HCl and NaOH. In the pharmaceutically relevant case of acidic glucagon, the removal of aggregates by filtration significantly slowed the aggregation process. Field-flow fractionation was used to separate aggregates from monomeric glucagon and determine relative mass. The molar mass of the large aggregates was shown to grow appreciably over time as the glucagon solutions gelled.

**Conclusion:**

The results of this study indicate that initial glucagon solutions are predominantly monomeric, but contain small quantities of large aggregates. These results suggest that the initial aggregates are seed nuclei, or intermediates which catalyze the aggregation process, even at low concentrations.

## Background

Glucagon is a 29-residue peptide hormone involved in the regulation of blood glucose. Glucagon has several uses as a therapeutic agent, including the emergency treatment of hypoglycemia [1]. Pharmaceutical preparations of glucagon are formulated in the amorphous solid state, and must be solubilized immediately prior to administration. Once in solution, glucagon is physically unstable and must be discarded after 24 hours due to gel formation. In dilute acid, the medium in which lyophilized glucagon is normally solubilized, this gel formation has been shown to result from the growth of fibrillar aggregates [2]. Protein aggregation is a problem in the manufacture, formulation, and delivery of biopharmaceutical products like glucagon. The presence of aggregates can result in reduced biological activity, and other complications with parenteral delivery, including increased immunogenicity [3]. Understanding the aggregation process is important not only for the pharmaceutical production and therapeutic use of glucagon, but also for elucidating a general mechanism of fibril formation.

Much remains to be learned about the mechanism by which proteins associate into amyloid fibrils, the characteristic feature of over 20 degenerative conditions [4]. The toxic species in such diseases has been identified as the intermediates in the aggregation pathway rather than the insoluble, mature fibrils and plaques [5,6], although the exact molecular mechanism of pathogenesis is controversial [7]. Protein aggregation has been described as a nucleation-dependent process, specifically that the aggregation rate can be seeded by the addition of intermediate aggregates [8]. In general, determining the mechanism for fibril formation at the molecular level may be the key to understanding the basis for amyloid toxicity and disease prevention.

Glucagon has been presented as an ideal model for characterizing fibril formation, since the aggregation process can be studied at pharmaceutically and clinically relevant conditions [9]. Recently, the structure of glucagon fibrils has been probed extensively by atomic force microscopy (AFM) [9-12] as well as electrophoretic and spectroscopic techniques[13]. Various solution conditions have been shown to result in at least two different types of mature fibrils. However, the small oligomeric precursors which serve to initiate and nucleate the process remain relatively uncharacterized. In this study, these initial aggregates, or seed nuclei, are characterized in bulk solution using light scattering methods. The advantages of light scattering techniques over other methods for studying fibril formation are that light scattering experiments are non-invasive to the sample, and can provide absolute determination of hydrodynamic size and molar mass. Unlike AFM, protein aggregates are examined by light scattering in the bulk liquid phase rather than as deposited or adsorbed species on a solid substrate, where the growth and morphology of aggregates may be quite different. Static and dynamic light scattering have been used to monitor the aggregation process of fibril-forming proteins such as β-amyloid [14,15], α-synuclein [16], and huntingtin [17].

Traditionally, polydisperse protein solutions have been separated by size-exclusion chromatography (SEC) for downstream light scattering analysis. In a recent study, the size of glucagon aggregates was evaluated by SEC [18]. However, viscous gel-like protein aggregates have been known to cause plugging and fouling problems in chromatography columns, often leading to irreproducible results. For this reason, the separation method of field-flow fractionation (FFF) has been employed in this study. This technology is unique in that it can be used to separate materials over a much broader range of particle sizes than traditional analytical methods, from 1 nm up to 100 μm. Separation in FFF takes place in an open flow channel, greatly reducing shear forces due to the absence of stationary phase. Asymmetric flow FFF can be coupled with multi-angle laser light scattering for molar mass and size determination independent of molecular weight standards. This combination of analytical methods has recently been used to characterize polysaccharides [19], water-soluble polymers [20], and nanosized drug carrier systems [21,22]. However, it has not been widely used protein applications. In this study, FFF is shown to be an effective method for separating intermediate aggregates from monomeric glucagon.

### Static light scattering theory

In static light scattering (SLS) experiments, the time-averaged intensity of scattered light is measured as a function of particle concentration and scattering angle. These measurements yield the weight-averaged molar mass (*M*_*w*_) of the scattering particles. If the size of the scattering species is smaller than *λ*/20 it will behave as a point source. In this case, a simplified form of the Debye equation, based on Rayleigh-Gans-Debye theory, can be applied for vertically polarized light,

(1) $$\frac{Kc}{R_{\theta }}=\frac{1}{M_{w}}+2B_{22}c$$

where

(2) $$K=\frac{4\pi^{2}{\left[n_{0}(dn/dc)\right]}^{2}}{N_{A}\lambda^{4}},$$

*c *is the concentration of the scattering species in solution, *R*_*θ *_is the Rayleigh ratio or the excess intensity of scattered light at *θ *scattering angle, *N*_*A *_is Avogadro's number, λ is the wavelength of the laser light source, *n*_0 _is the refractive index of the solvent, and *B*_22 _is the second osmotic virial coefficient of the scattering species.

The *M*_*w *_is determined by plotting values of *Kc*/*R*_*θ *_versus particle concentration at a given scattering angle. This graphical representation is referred to as a Debye plot. The y-intercept determined through linear regression gives the reciprocal weight-averaged molar mass (1/*M*_*w*_) of particles in solution.

### Dynamic light scattering theory

Dynamic light scattering (DLS) measures fluctuations in the intensity of scattered light which arise from thermal motion of particles in the system. Analysis of these temporal fluctuations allows for estimation of the diffusion coefficient, *D*, which can be used to determine the apparent hydrodynamic radius (*R*_*h*_) and particle size distribution (PSD) of the scattering species. The rate at which the scattered light fluctuates is directly related to the "speed" at which molecules move due to diffusion. The time autocorrelation function of the scattered light intensity *G*_2_*(t) *provides a quantitative measure of the particle motion. Most theory is developed using the scattered electric field autocorrelation function *g*_1_*(t)*, which is easily calculated from the experimentally measured light intensity autocorrelation function by use the Siegert relationship,

(3) $$g_{1}\left(t\right)={\left[\frac{G_{2}\left(t\right)-G_{2}\left(\infty \right)}{G_{2}\left(\infty \right)}\right]}^{\frac{1}{2}}$$

where *G*_2_*(∞) *is the baseline of the experimentally measured intensity autocorrelation function.

For non-interacting, monodisperse particles that are small compared with the wavelength of light, the scattered electric field correlation function g_1_(t) decays exponentially,

(4) *g*_1_(*t*) = exp(-*t*/*τ*)

where τ is the relaxation time of the scattered field correlation function, and can be related to the diffusion coefficient of the scattering species

(5) $$\tau =\frac{1}{Dq^{2}}$$

with scattering vector, *q*, given by

(6) *q *= (4*πn*_0_/*λ*) sin (*θ*/2)

The apparent *R*_*h *_can be determined using the Stokes-Einstein relationship, which relates the diffusion coefficient of a hard sphere at infinite dilution (*D*_0_) to its hydrodynamic radius

(7) $$D_{0}=\frac{kT}{6\pi \eta R_{h}}$$

where *k *is the Boltzmann constant and *η *is the solvent viscosity at absolute temperature, *T*. This simplified analysis ignores concentrative effects due to hydrodynamic and thermodynamic interactions which have been described elsewhere.

Information about relaxation time and apparent hydrodynamic radius distributions can be extracted from the data by regressing appropriate theoretical models to the autocorrelation function. For example, if a bimodal distribution is expected, a two-exponential fit is performed estimate the two relaxation times independently.

(8) *g*_1_(*t*) = *a*_1 _exp(-*t*/*τ*_1_) + *a*_2 _exp(-*t*/*τ*_2_)

Parameters *a*_1 _and *a*_2 _are expansion coefficients known as light intensity weighted amplitudes and *τ*_1 _and *τ*_2 _are relaxation times.

Generalizing from this approach to systems having multiple particle sizes or broad size distributions is straightforward in principle, but requires specialized computational approaches in practice. Each scattering species can be represented by a corresponding relaxation time, resulting in a sum of exponentials

(9) *g*_1_(*t*) = *a*_1 _exp(-*t*/*τ*_1_) + *a*_2 _exp(-*t*/*τ*_2_)+...

where *a*_*n *_values represent the relative contributions (amplitudes) of each particle size. The regularized inverse Laplace transform program *CONTIN *is routinely utilized to perform such multi-exponential analyses of relaxation-time distributions [23]. Under the assumption that the scattering particles behave as hard spheres in dilute solution, the relaxation time distribution obtained can be converted into an apparent hydrodynamic size distribution using the Stokes-Einstein relationship.

### FFF/MALS theory

The theory and principles of asymmetric flow FFF have been extensively reviewed elsewhere [24-26]. In summary, this type of FFF is performed inside a thin, ribbon-like channel approximately 30 cm in length, 2 cm wide, and ranging in thickness up to 500 μm. Carrier fluid is pumped through the channel from the inlet end exhibiting a laminar flow profile. A cross-flow is induced perpendicular to the channel flow, which exits the channel through the bottom wall fitted with an ultrafiltration membrane. The cross-flow forces the sample components toward this "accumulation wall" of the channel, where a concentration gradient is established. Small particles with higher diffusion coefficients achieve equilibrium positions at higher levels in the channel than larger particles, and are thus transported through the channel more rapidly due to the parabolic profile of the channel flow. Consequently, small particles elute first, opposite the order of elution in SEC.

In the manner described above, FFF accomplishes the separation of different sized particles in polydisperse solutions for analysis by downstream detectors. Molar masses of the fractionated species are determined using a multi-angle light scattering (MALS) detector which collects on-line SLS measurements at multiple scattering angles. The linear Zimm method [27] is used to obtain molar mass by setting *B*_22 _to zero and extrapolating to zero scattering angle.

## Methods

### Preparation of Protein Standards for FFF/MALS Method Validation

The following protein standards were obtained in lyophilized powder form from Sigma Aldrich (St. Louis, MO): bovine serum albumin (BSA), alcohol dehydrogenase, β-amylase, apoferritin, and thyroglobulin. All protein standards were dissolved in Dulbecco's phosphate buffer solution (PBS), pH 6.5, also obtained in powder form from Sigma and dissolved in chromatography grade Optima water from Fisher Scientific (Fair Lawn, NJ). PBS was filtered with 0.1 μm filters before use.

### Preparation of Glucagon Solutions

**Figure 1:**
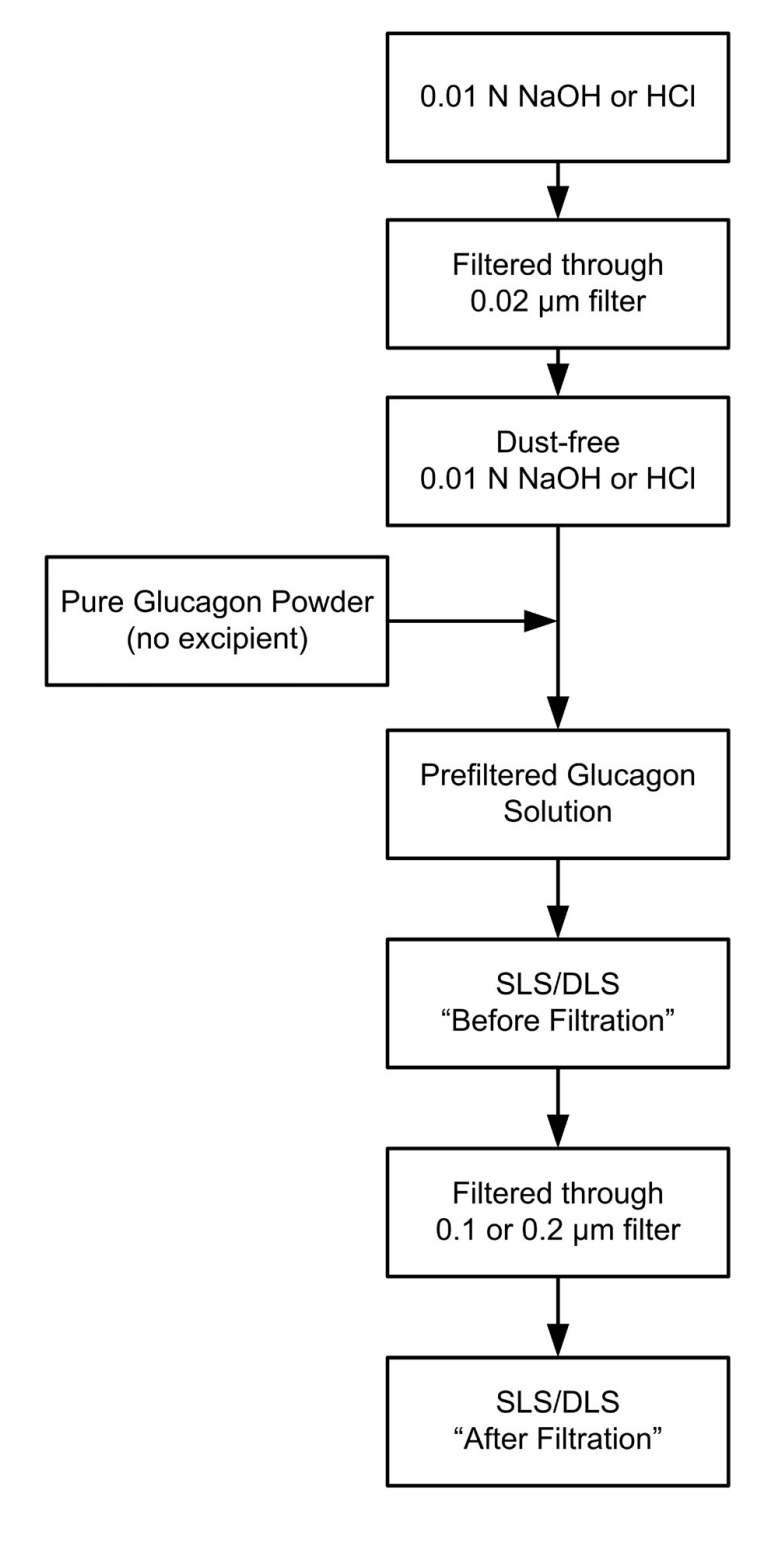
Process diagram depicting preparation of solvents and glucagon solutions.

Lyophilized glucagon powder was donated by Eli Lilly and Company (Indianapolis, IN). Solvents used were certified 0.01 N HCl or 0.01 N NaOH purchased from Fisher Scientific (Fair Lawn, NJ). Prior to the addition of glucagon, the small quantities of solvents used in these studies were filtered through 0.02 μm syringe filters. This step is necessary in order to produce dust-free diluents required for light scattering studies. The glucagon powder was then dissolved in the dust-free HCl or NaOH. Light scattering experiments were performed on the glucagon solutions, both before and after an additional filtration using either 0.1 or 0.2 μm solvent filters obtained from Millipore (Bedford, MA) and Whatman (Kent, UK). A process diagram detailing the solvent and glucagon solution preparations is shown in Figure 1. Glucagon concentrations were determined spectrophotometrically using *E *(1 mg/mL; 1 cm) = 2.38 at 278 nm.

### SLS and DLS measurements

SLS and DLS experiments were conducted with an ALV-GmbH (Langen, Germany) SP-125 Compact DLS/SLS Goniometer. The majority of experiments presented here utilized a vertically polarized 400 mW diode-pumped, solid-state Coherent DPSS532-400 laser (Coherent Inc., Santa Clara, CA) operating at 532 nm wavelength as the light source. Glucagon solutions were prepared as previously described, placed in the sample compartment, fixed to restrict movement, and equilibrated at 30°C by a thermostatted bath. In DLS experiments, the apparent hydrodynamic size and PSD of glucagon samples were determined by obtaining autocorrelation functions of the scattered light intensity, *G*_2_*(t)*, using an ALV-5000/E multiple tau digital correlator. Time correlation functions were analyzed using *CONTIN *software to determine *R*_*h *_and PSD. The specific refractive index (d*n*/d*c*) of glucagon was measured using a Bellingham & Stanley (Kent, UK) 60/ED Abbe refractometer with the laser light source described. The values obtained for d*n*/d*c *at 532 nm were 0.175 in HCl and 0.189 in NaOH.

### FFF/MALS

Asymmetric flow FFF experiments were carried out with an Eclipse F separation system from Wyatt Technology Corporation (WTC, Santa Barbara, CA). For protein standard analysis, 250 μm channel thickness and 10 kDa MWCO membranes were used, also obtained from WTC. Protein standards were prepared in PBS as previously described, and this buffer was used as the mobile phase. For glucagon experiments, 450 μm channel thickness and 1 kDa MWCO ultrafiltration membranes were used, obtained from WTC. Glucagon solutions were prepared in certified 0.01 N HCl as previously described, and this solvent was used as the mobile phase. In all experiments, samples were injected into the FFF channel using a manual Rheodyne (Rohnert Park, CA) injection port. Channel flow was set at 1 mL/min, and cross-flow rates were controlled using the Eclipse2 software, version 2.3 (WTC).

FFF experiments were coupled with MALS detection for molar mass determination. The MALS detector used was an 18-angle DAWN EOS (WTC), which employs a 685 nm wavelength 30 mW linearly polarized Ga-As laser light source. Molar mass calculations were made using ASTRA software (WTC). On-line concentration measurements were made with a Shimadzu (Columbia, MD) SPD-10AV UV spectrophotometer set at a wavelength of 278 nm to monitor the absorbance of light by glucagon.

## Results and Discussion

### Validation of FFF/MALS method for protein characterization

**Figure 2:**
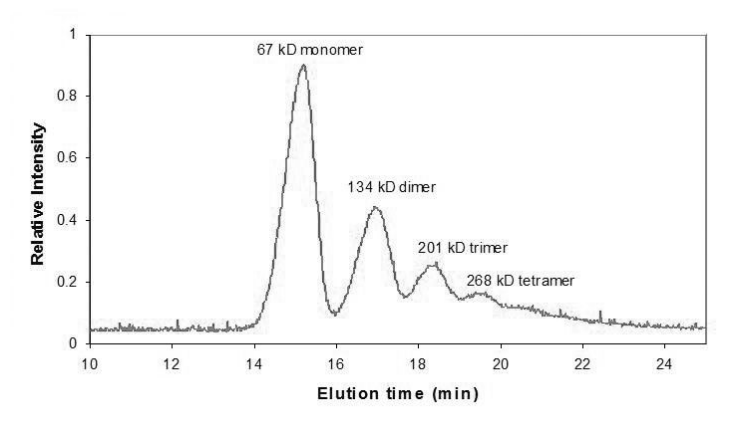
Relative light scattering intensity vs. elution time for optimal separation of BSA oligomers by FFF/MALS.

Separation of BSA oligomers was demonstrated using the FFF/MALS method described, in order to validate this method as suitable for protein separation. BSA is a common protein with a monomer molecular weight of 67 kDa that is known to form well defined oligomers in solution. This experiment is routinely used to optimize FFF performance. Figure 2 shows an optimal BSA separation, with 70 μg of BSA injected using PBS as mobile phase. The relative light scattering intensity of BSA monomer, dimer, trimer, tetramer and higher order aggregates can be identified.

**Figure 3:**
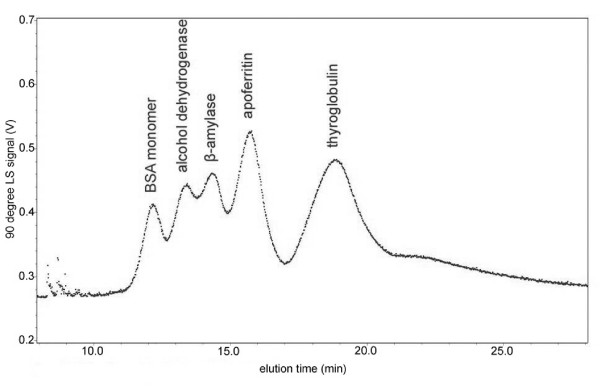
Separation of protein standards by FFF/MALS showing 90 degree light scattering signal vs. elution time.

In order to demonstrate the ability of FFF/MALS to not only speciate aggregates, but also to provide absolute molecular weight determination, a mixture of five different protein standards was analyzed: BSA, alcohol dehydrogenase, β-amylase, apoferritin, and thyroglobulin. The light scattering signal for the five separated proteins is shown in Figure 3. The injected quantity of each protein is shown in Table 1, along with a comparison between expected molecular weights and the *M*_*w *_inferred from the light scattering results. The results of these FFF/MALS experiments using protein standards demonstrate that FFF is an effective method for separating proteins and protein aggregates.

**Table 1:** Protein standards expected MW compared with *M*_*w *_inferred from FFF/MALS experiments

Protein Standard	Injected amount (μg)	Expected MW (kDa)	*M*_*w *_inferred from light scattering (kDa)
BSA monomer	42.5	67	62.8
alcohol dehydrogenase	59.2	150	137.4
β-amylase	20.8	200	202.8
apoferritin	28.8	443	440.1
thyroglobulin	58.3	669	675.2

### Presence of high molecular weight glucagon aggregates

**Figure 4:**
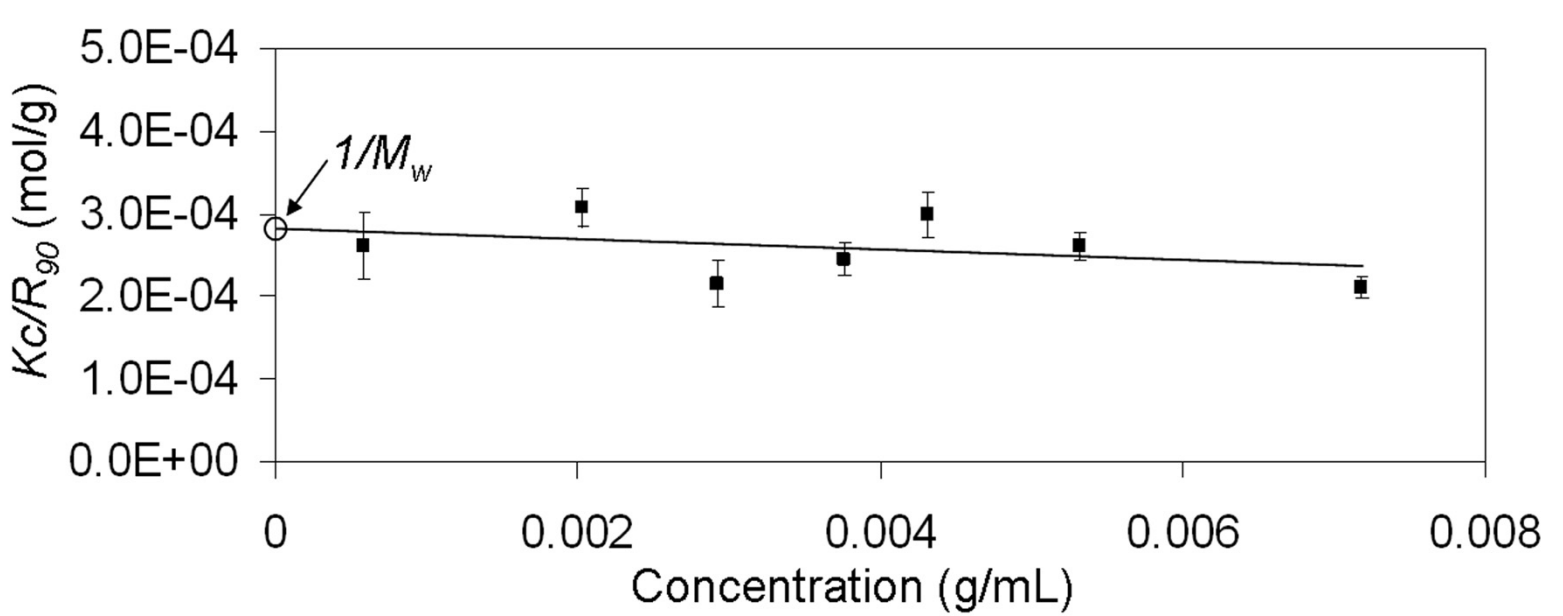
**Debye plot of glucagon immediately upon dissolving in 0.01 N HCl. **Measurements obtained by static light scattering at 30°C and a 90° scattering angle. Linear regression model generated using *Kc/R*_90 _and glucagon concentration yielded a y-intercept of 2.8 (± 0.3) × 10^-4^, which corresponds to a *M*_*w *_= 3539 (± 372).

**Figure 5:**
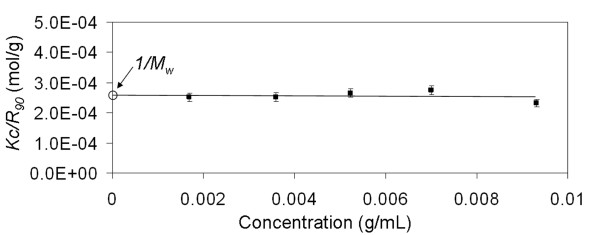
**Debye plot of glucagon immediately upon dissolving in 0.01 N NaOH. **Measurements obtained by static light scattering at 30°C and a 90° scattering angle. Linear regression model generated using *Kc/R*_90 _and glucagon concentration yielded a y-intercept of 2.6 (± 0.2) × 10^-4^, which corresponds to a *M*_*w *_= 3831 (± 275).

Filtered glucagon solutions were analyzed immediately after mixing by SLS to determine the weight-averaged molar mass (*M*_*w*_) using the Debye equation (Eqn. 1). The excess Rayleigh ratio at 90° scattering angle, *R*_90_, was measured over a range of known concentrations. By plotting values of *Kc*/*R*_90 _over a range of concentrations, the *M*_*w *_was determined from the inverse of the y-intercept value. Debye plots of *Kc/R*_90 _as a function of concentration for acidic and alkaline glucagon solutions are shown in Figure 4 and Figure 5. The *M*_*w *_of soluble glucagon in the acidic pH region was determined to be 3539 ± 372 g/mol. Glucagon in alkaline solutions exhibited a slightly higher *M*_*w*_, 3831 ± 275 g/mol. This slightly higher alkaline *M*_*w *_may indicate the presence of higher molecular weight aggregates in the alkaline solutions. In order to verify this, DLS was utilized to further probe these samples.

**Figure 6:**
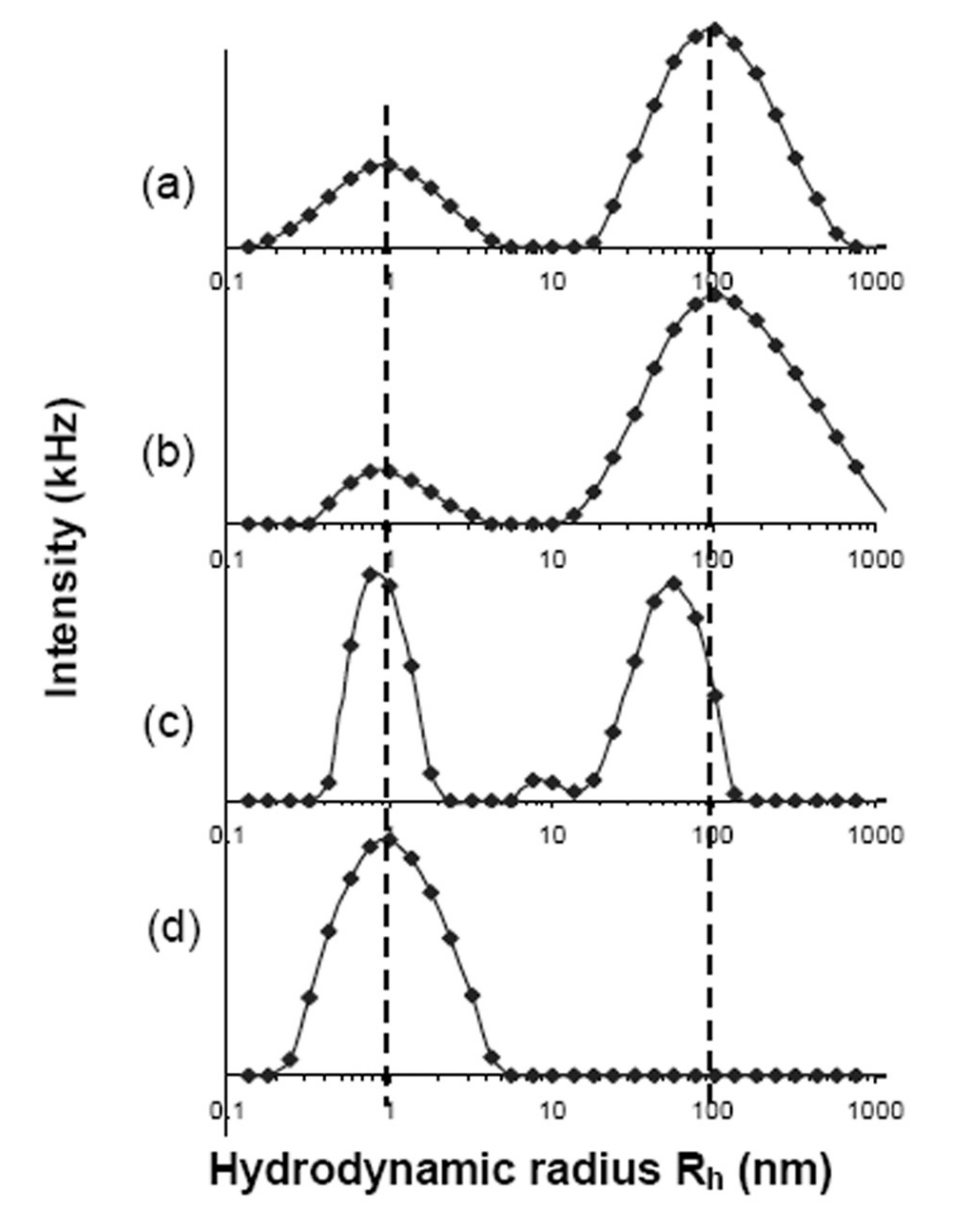
**Hydrodynamic size distributions obtained from relaxation time distributions corresponding to the autocorrelation functions of unfiltered and filtered glucagon solutions.** (a) and (b) represent the PSD of unfiltered glucagon in 0.01 N NaOH and HCl, respectively, (c) and (d) represents 0.2 μm filtered alkaline and acid glucagon solutions, respectively.

Relaxation time distributions before and after filtration for both acidic and alkaline glucagon were obtained from *CONTIN *analysis of light intensity autocorrelation functions. The particle *R*_*h *_distribution functions were calculated from the relaxation time distributions using the Stokes-Einstein relationship. *CONTIN *analysis generated *R*_*h *_distribution plots of unfiltered acidic and alkaline glucagon solutions are shown in Figure 6a and 6b. These unfiltered glucagon solutions in dilute acid and base showed bimodal *R*_*h *_distributions with peak *R*_*h *_values around 1 and 100 nm, with the 1 nm peak indicating monomeric glucagon. The monomeric radius of glucagon can be confirmed using an accepted equation for approximating the volume of a typical monomeric protein from the *M*_*w*_[28]. Using a literature reported molecular weight of 3485 g/mol, which is based on the amino acid sequence of glucagon, the estimated monomeric radius is 1.02 nm.

### Removal of aggregates in acidic glucagon solutions slows gelation rate

DLS results indicate that acidic and alkaline glucagon solutions exhibit distinctly different behavior following filtration. Bimodal distributions were still present following the filtration of alkaline glucagon solutions (Fig. 6c), whereas the higher molecular weight aggregates were eliminated by filtration of acidic glucagon solutions (Fig. 6d). The bimodal distribution that remained after filtration of alkaline glucagon solutions showed the larger subpopulation peak hydrodynamic radius value shifting to a slightly smaller *R*_*h *_of 61 nm from its original size of 100 nm.

These DLS results indicate the presence of higher molecular weight aggregates that can be removed by filtration from acidic but not alkaline solutions. The filter retention may be related to the relative charge of the filter membrane or to the relative rigidity of the aggregates. Since the filters used were designed to have low affinity for protein, the aggregate rigidity seems to be the more plausible explanation for the observed effects.

**Figure 7:**
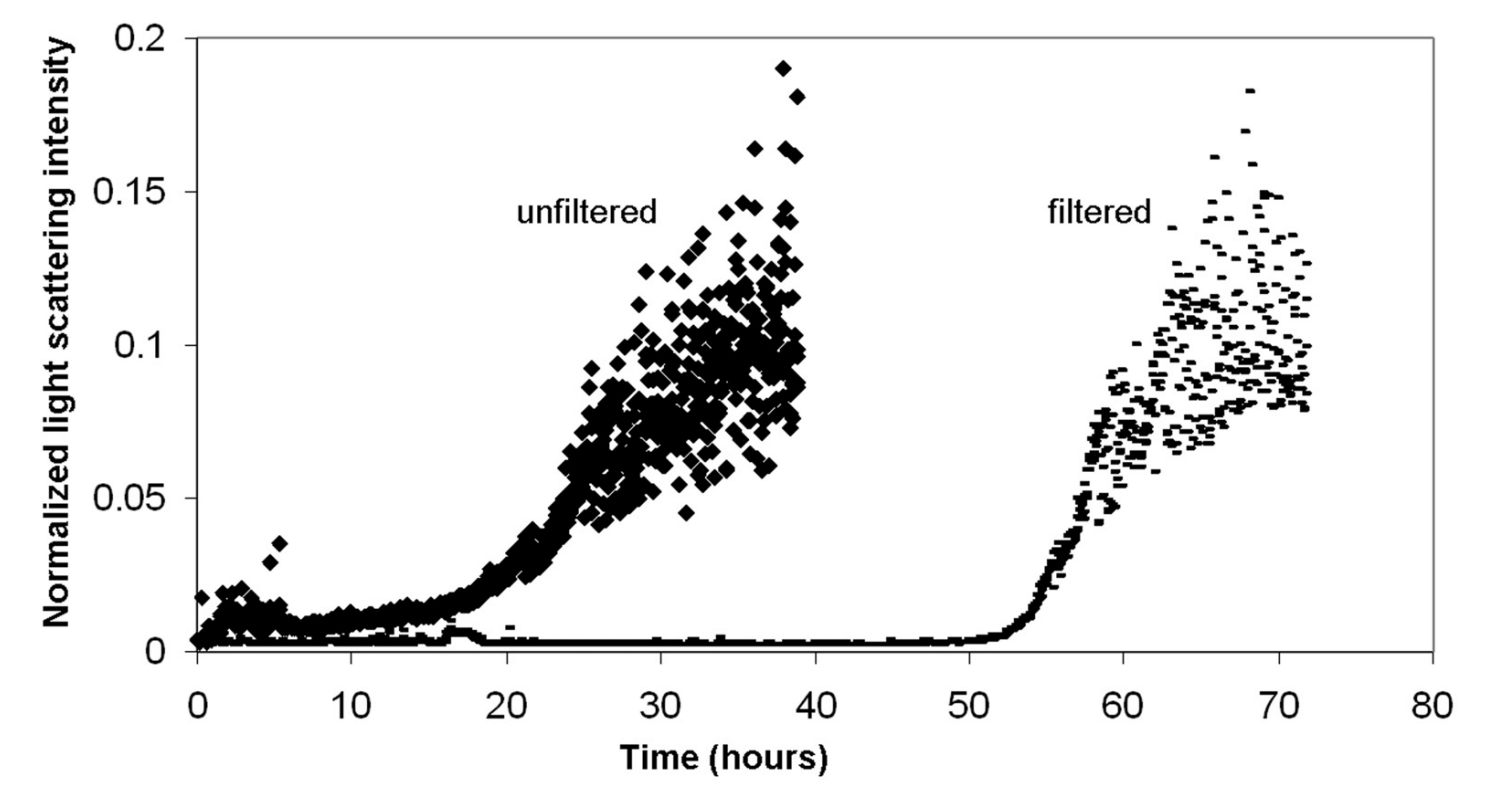
Raw scattered light intensity from ALV SLS detector vs. time for filtered and unfiltered glucagon preparations in dilute HCl.

The effect of pre-existing aggregates on aggregation rate was further investigated by measuring the raw light scattering signal intensity of freshly prepared 4 mg/mL glucagon solutions in 0.01 N HCl over a period of three days (Fig. 7). The rapid increase in light scattering intensity for the unfiltered glucagon preparation suggests that the presence of initial aggregates serve to nucleate the aggregation process. Conversely, the filtered acid glucagon preparation with the absence of initial aggregates stayed stable in solution for 50 hours.

### Characterization of glucagon aggregates by FFF/MALS

To further quantify the two subpopulations within the unfiltered samples, SEC experiments were attempted. SEC proved ineffective in monitoring the molecular weight distribution of aggregating glucagon systems (data not shown). Aggregates present in glucagon solutions injected into a column using 0.01 N HCl eluent caused fouling and backpressure problems in the SEC system, and tended to dissociate inside the column, emerging in the monomeric state. Thus, this method was abandoned as a means to monitor the aggregation process.

**Figure 8:**
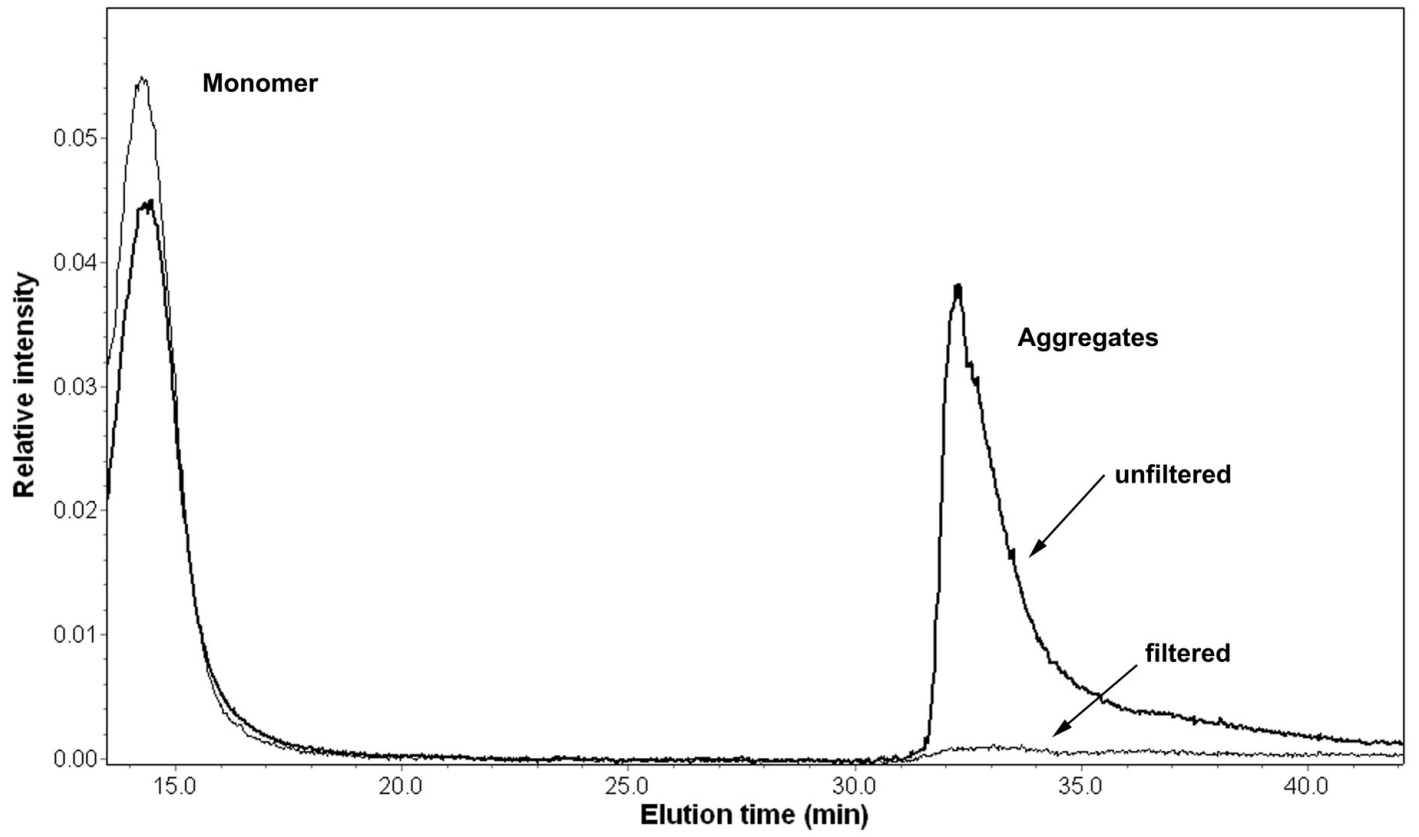
**Relative intensity of Rayleigh ratio from MALS detector for filtered and unfiltered glucagon solutions in 0.01 N HCl.** Monomer and aggregates have been separated by asymmetric flow FFF in the unfiltered sample. Aggregates have been removed from the solution in the filtered sample.

FFF/MALS experiments were performed to verify the presence of large initial aggregates upon initial mixing of glucagon in 0.01 N HCl, and to confirm that the large aggregates can be removed by filtration, as previously indicated by DLS results. The 100 nm aggregate population was not concentrated enough to be detectable at the low concentrations and small injection volumes typically used with chromatographic methods. Therefore 250 μL of a 14 mg/ml glucagon solution was injected into the FFF channel for these experiments both before and after filtration with a 0.1 μm filter. Elution of a large aggregate peak can be observed in the unfiltered sample, but not in the filtered sample (Fig. 8). The use of a UV detector downstream of the FFF unit allowed for the verification that the large particles are indeed protein, since this peak exhibits measurable UV absorbance at 278 nm.

**Figure 9:**
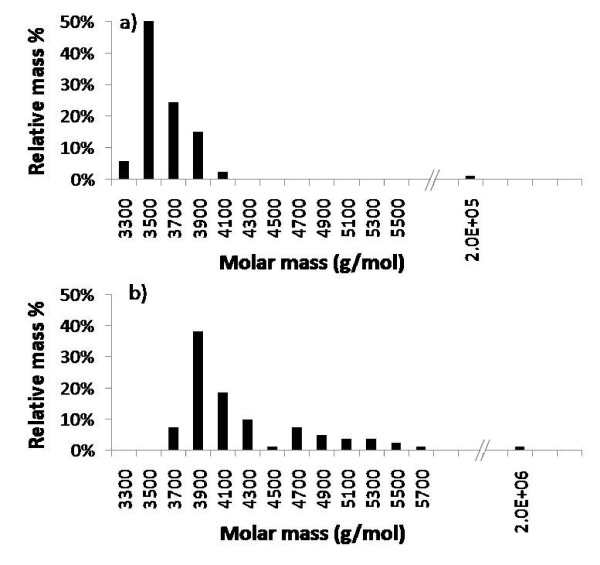
**Histograms showing molar mass distributions for glucagon monomer and unfiltered aggregates analyzed by FFF/MALS.** Initial glucagon solution evaluated immediately after dissolving in 0.01 N HCl is shown in a). Molar mass distribution after 22 hours incubation is shown in b). Molar mass of the large aggregate has grown by one order of magnitude over this period of time.

A high concentration unfiltered glucagon solution in 0.01 N HCl was again analyzed by FFF, both before and after 22 hours of incubation at 22°C. Initially, weight averaged molar mass of the monomer was analyzed by MALS to be 3650 g/mol, with a large aggregate peak of 1.2 × 10^5 ^g/mol. After the incubation period, the sample was observed to be partially gelled. When this sample was again analyzed by FFF/MALS, the molar mass of the large aggregate had increased significantly to 1.9 × 10^6 ^g/mol. The histograms in Figure 9 show the relative molar mass distributions for the two cases. The large aggregates are on the order of 1% of total injected mass, but the molar mass distributions for these peaks cannot be further analyzed due to extremely low UV signal strength.

## Conclusion

These results indicate that initial glucagon solutions are predominantly monomeric, but contain small quantities of large aggregates. Although the concentration of these initial aggregates is low, these particles serve to catalyze or nucleate new aggregates. Separating and characterizing the large particles which absorb UV at 278 nm by FFF suggests that these particles are aggregates of glucagon that grow over time. Thus, further aggregation of glucagon is accelerated by these seed nuclei, by a process such as

(10) $$nM\overset{catalyzed\;by\;M_{n}}{\to }M_{n}$$

where *nM *represents *n *glucagon monomers, and *M*_*n *_represents an oligomeric species of number n. It was demonstrated that filtration can remove the large aggregates in some situations.

These findings describe an important aspect of the aggregation behavior of glucagon, which is considered a model amyloidgenic protein. This work contributes to the field of biological engineering because of its relevance to drug formulation and delivery, as well as clinical applications.

## Competing interests

The authors declare that they have no competing interests.

## Authors' contributions

CH carried out SLS, DLS, and FFF experiments. LN carried out SLS and DLS experiments. CH, JW, and LN all contributed to the writing of the manuscript. JW and LK conceived of the study, and participated in its design and coordination. All authors read and approved the final manuscript.

## References

[B1] Kim DY, Shin NK, Chang SG, Shin HC (1996). Production of recombinant human glucagon in *Escherichia coli* by a novel fusion protein approach. Biotechnology Techniques.

[B2] Beaven GH, Gratzer WB, Davies HG (1969). Formation and structure of gels and fibrils from glucagon. Eur J Biochem.

[B3] Costantino HR, Langer R, Klibanov AM (1994). Solid-phase aggregation of proteins under pharmaceutically relevant conditions. J Pharm Sci.

[B4] Stefani M (2004). Protein misfolding and aggregation: new examples in medicine and biology of the dark side of the protein world. Biochim Biophys Acta.

[B5] Bucciantini M, Giannoni E, Chiti F, Baroni F, Formigli L, Zurdo JS, Taddei N, Ramponi G, Dobson CM, Stefani M (2002). Inherent toxicity of aggregates implies a common mechanism for protein misfolding diseases. Nature.

[B6] Kirkitadze MD, Bitan G, Teplow DB (2002). Paradigm shifts in Alzheimer's disease and other neuro degenerative disorders: The emerging role of oligomeric assemblies. J Neurosci Res.

[B7] Stefani M, Dobson CM (2003). Protein aggregation and aggregate toxicity: new insights into protein folding, misfolding diseases and biological evolution. J Mol Med.

[B8] Koo EH, Lansbury PT, Kelly JW (1999). Amyloid diseases: Abnormal protein aggregation in neurodegeneration. Proc Natl Acad Sci USA.

[B9] De Jong KL, Incledon B, Yip CM, DeFelippis MR (2006). Amyloid fibrils of glucagon characterized by high-resolution atomic force microscopy. Biophys J.

[B10] Dong MD, Hovgaard MB, Xu SL, Otzen DE, Besenbacher F (2006). AFM study of glucagon fibrillation via oligomeric structures resulting in interwoven fibrils. Nanotechnology.

[B11] Pedersen JS, Dikov D, Flink JL, Hjuler HA, Christiansen G, Otzen DE (2006). The changing face of glucagon fibrillation: Structural polymorphism and conformational imprinting. J Mol Biol.

[B12] Pedersen JS, Flink JM, Dikov D, Otzen DE (2006). Sulfates dramatically stabilize a salt-dependent type of glucagon fibrils. Biophys J.

[B13] Onoue S, Iwasa S, Kojima T, Katoh F, Debari K, Koh K, Matsuda Y, Yajima T (2006). Structural transition of glucagon in the concentrated solution observed by electrophoretic and spectroscopic techniques. J Chromatogr A.

[B14] Pallitto MM, Murphy RM (2001). A mathematical model of the kinetics of beta-amyloid fibril growth from the denatured state. Biophys J.

[B15] Lomakin A, Teplow DB, Kirschner DA, Benedek GB (1997). Kinetic theory of fibrillogenesis of amyloid beta-protein. Proc Natl Acad Sci USA.

[B16] Li J, Uversky VN, Fink AL (2002). Conformational behavior of human alpha-synuclein is modulated by familial Parkinson's disease point mutations A30P and A53T. Neurotoxicology.

[B17] Georgalis Y, Starikov EB, Hollenbach B, Lurz R, Scherzinger E, Saenger W, Lehrach H, Wanker EE (1998). Huntingtin aggregation monitored by dynamic light scattering. Proc Natl Acad Sci USA.

[B18] Onoue S, Ohshima K, Debari K, Koh K, Shioda S, Iwasa S, Kashimoto K, Yajima T (2004). Mishandling of the therapeutic peptide glucagon generates cytotoxic amyloidogenic fibrils. Pharm Res.

[B19] Wittgren B, Borgstrom J, Piculell L, Wahlund KG (1998). Conformational change and aggregation of kappa-carrageenan studied by flow field-flow fractionation and multiangle light scattering. Biopolymers.

[B20] Viebke C, Williams PA (2000). The influence of temperature on the characterization of water-soluble polymers using asymmetric flow field-flow-fractionation coupled to multiangle laser light scattering. Anal Chem.

[B21] Fraunhofer W, Winter G, Coester C (2004). Asymmetrical flow field-flow fractionation and multiangle light scattering for analysis of gelatin nanoparticle drug carrier systems. Anal Chem.

[B22] Arifin DR, Palmer AF (2003). Determination of size distribution and encapsulation efficiency of liposome-encapsulated hemoglobin blood substitutes using asymmetric flow field-flow fractionation coupled with multi-angle static light scattering. Biotechnol Prog.

[B23] Provencher SW (1979). Inverse problems in polymer characterization - direct analysis of polydispersity with photon correlation spectroscopy. Makromol Chem.

[B24] Giddings JC (1968). Nonequilibrium theory of field-flow fractionation. J Chem Phys.

[B25] Wahlund KG, Giddings JC (1987). Properties of an asymmetrical flow field-flow fractionation channel having one permeable wall. Anal Chem.

[B26] Fraunhofer W, Winter G (2004). The use of asymmetrical flow field-flow fractionation in pharmaceutics and biopharmaceutics. Eur J Pharm Biopharm.

[B27] Wyatt PJ (1993). Light scattering and the absolute characterization of macromolecules. Anal Chim Acta.

[B28] Creighton TE (1993). The general properties of protein structures. Proteins: Structures and Molecular Properties.

